# Predicting MCI-to-Alzheimer’s Conversion: A Systematic Benchmark of Classical, Machine Learning, and Deep Survival Models

**DOI:** 10.3390/diagnostics16203321

**Published:** 2026-10-13

**Authors:** Nathalia Arias Santa, Genaro Daza-Santacoloma, Walter Serna Serna

**Affiliations:** Automatics Research Group, Department of Engineering, Universidad Tecnológica de Pereira (UTP), Pereira 660003, Colombia; genaro.santacoloma@utp.edu.co (G.D.-S.); wserna@utp.edu.co (W.S.S.)

**Keywords:** Alzheimer’s disease, mild cognitive impairment, survival analysis, time-to-event prediction, dynamic risk prediction, serial neuroimaging, biomedical signal processing, machine learning, deep learning, ADNI, OASIS-3, independent-cohort analysis

## Abstract

**Background/Objectives:** Predicting time-to-conversion from mild cognitive impairment (MCI) to Alzheimer’s disease (AD) is a critical clinical challenge. Most survival analyses rely on static baseline variables, ignoring the temporal dynamics of serial neuroimaging. We benchmark seven survival model families using a novel temporal MRI descriptor pipeline on ADNI. **Methods:** A cohort of 661 MCI patients (250 conversion events, 37.8%, median follow-up 42.0 months) was constructed from ADNI. FreeSurfer volumetrics of six brain regions were summarised into three time-based descriptors each (atrophy rate, trajectory variability σ, and time-weighted cumulative mean μ) under strict anti-leakage constraints, yielding 18 neuroimaging features which, combined with six clinical variables (age, sex, education, MMSE, ADAS-13, and APOE-ε4), form a 24-dimensional covariate vector. Cox PH, Weibull AFT, Exponential, RSF, GBST, DeepSurv, and DeepHit were evaluated under stratified 5-fold cross-validation and assessed on an independent OASIS-3 cohort (n=78) as an exploratory analysis. **Results:** All seven models achieved C-index 0.853–0.863 (Δ=0.010), with Cox PH reaching 0.863±0.027 using only routine structural MRI. Time-dependent AUC ranged from 0.856 (12 months) to 0.938 (48 months). Risk stratification by quartile yielded highly separated Kaplan-Meier curves (log-rank p<0.001). On OASIS-3 (n=78, exploratory), classical models showed preliminary cross-cohort consistency (Weibull AFT 0.760±0.063, RSF 0.759±0.049), while deep learning degraded substantially (DeepSurv 0.551±0.116); these results are hypothesis-generating and should not be interpreted as confirmatory given the small per-fold sample size (EPV ≈ 1). **Conclusions:** The temporal MRI descriptor pipeline achieves a C-index of 0.863 using only routine structural MRI, without PET or CSF biomarkers, demonstrating strong discriminative performance on ADNI MCI-to-AD conversion. The narrow performance band (Δ=0.010) suggests that, within this feature-engineering paradigm, no clear performance separation was observed among the evaluated model architectures; this convergence may reflect feature-level rather than architecture-level constraints, although the underlying cause cannot be established from discriminative performance alone. Classical models showed more stable cross-cohort behaviour under data-scarce conditions; prospective validation on larger cohorts with full covariate parity is required before clinical translation can be considered.

## 1. Introduction

Alzheimer’s disease (AD) is a progressive neurodegenerative disorder and the leading cause of dementia worldwide, currently affecting over 55 million individuals and projected to surpass 150 million by 2050 [1]. Mild Cognitive Impairment (MCI) represents the transitional clinical stage between normal cognitive aging and manifest AD, characterized by measurable cognitive deficits in the absence of functional impairment [1]. Approximately 10–15% of MCI patients convert to AD annually; however, individual trajectories are profoundly heterogeneous, with some patients converting within months while others remain stable for over a decade [2]. This heterogeneity underscores a critical clinical need: predicting not merely whether a patient will convert, but when, enabling timely initiation of disease-modifying therapies, improved stratification in clinical trials, and individualized patient monitoring [3].

To address this, it is necessary to use survival analysis, a statistical framework designed to model time-to-event outcomes. The main challenge posed by longitudinal clinical cohorts is right-censored data: a patient is considered censored when the event of interest is not observed within the study period. For example, a patient who remains stable at their final clinical evaluation is right-censored; we only know that conversion had not occurred by that point, not that it will never occur. Standard classification and regression methods cannot handle this partial temporal information, as they either exclude censored subjects or assign arbitrary event times, introducing systematic bias into the model estimates. Survival models address this directly by incorporating censored observations into their objective functions, using all available follow-up time to calculate unbiased hazard estimates [4].

For decades, the standard methodology for clinical risk prediction has been classical survival models, such as the Cox Proportional Hazards (CPH) model [5] and parametric models, including Weibull and Exponential regression [6]. These methods provide interpretable hazard ratios and good statistical performance under high censoring rates. However, their mathematical structure relies exclusively on static baseline measurements, making them unable to incorporate the temporal dynamics and non-linear trajectories that repeated-measures data encode [3,7]. This present work directly addresses that gap: by extracting structured time-based descriptors from the serial MRI measurements collected at each follow-up visit, we encode the evolving dynamics of brain atrophy into a fixed-length covariate vector that all seven survival model families can consume without modification. Importantly, because each patient’s descriptors are computed from all MRI sessions accumulated up to their event or censoring time ($t_{ij}\leq T_{i}$), the resulting pipeline constitutes a *dynamic risk prediction* framework: risk estimates are conditioned on the longitudinal history available at the time of assessment rather than on a fixed baseline snapshot. This design cannot generate predictions at the first clinical visit—it requires at least two to three serial MRI sessions—and is therefore most appropriately framed as dynamic prognostic estimation using accumulated longitudinal data. This approach is particularly well-suited to the Alzheimer’s Disease Neuroimaging Initiative (ADNI) [8], a large-scale longitudinal cohort in which each participant underwent repeated structural MRI scans at multiple clinical visits, typically every 6 to 12 months over a follow-up window of two to five years, providing the serial volumetric measurements whose temporal progression we explicitly exploit. Progressive morphological changes including hippocampal atrophy, entorhinal cortex thinning, and ventricular expansion are neurobiologically coupled with cognitive decline and conversion to AD [2]. Treating these repeated volumetric measurements as biomedical signals opens the possibility of applying formal signal processing and feature extraction techniques to capture rate-of-change dynamics that no single baseline value can represent. Throughout this work, the term temporal refers exclusively to the time dimension of serial measurements: temporal descriptors quantify how brain volumes evolve across successive MRI sessions over the follow-up period, and do not refer to the temporal lobe of the brain. This time-based perspective enables the extraction of structured features, namely atrophy rate, trajectory variability, and time-weighted cumulative mean, which encode the longitudinal progression of neurodegeneration in a form directly consumable by survival models.

To overcome the constraints of classical models, machine learning (ML) and deep learning (DL) have introduced powerful non-parametric alternatives for survival analysis. Ensemble methods such as Random Survival Forests (RSF) [9] and Gradient Boosting Survival Trees (GBST) [10] relax the proportional hazards assumption and learn non-linear covariate interactions without requiring explicit functional specifications. Deep neural network models, including DeepSurv [11] and DeepHit [12], extend these capabilities by learning hierarchical representations of covariates and optimizing loss functions specifically designed to handle censored observations, accommodating non-proportional and non-monotone risk dynamics. Despite these methodological advances, a critical gap persists in the literature: systematic and equitable benchmarks comparing classical, ML, and DL survival models under identical experimental conditions using serial neuroimaging signals as structured input remain scarce.

This study addresses these limitations through three principal contributions. First, a time-based signal representation pipeline is introduced that transforms serial MRI volumetric data from six anatomical brain regions into structured survival covariates. For each patient and region, three statistical descriptors are derived from the longitudinal trajectory of regional volumes: the atrophy rate (slope $\beta_{1}$, estimated by linear regression over the serial measurements), the trajectory variability (σ, standard deviation across visits), and the time-weighted cumulative mean (μ, a normalized integral of volume over follow-up time). This yields 18 neuroimaging features that encode the full temporal progression of brain atrophy in an interpretable and computationally efficient form. Second, a systematic multi-family survival benchmark is conducted encompassing seven time-to-event models of increasing complexity, namely classical parametric regression (Exponential, Weibull AFT, Cox PH), ensemble tree methods (RSF, GBST), and deep neural networks (DeepSurv, DeepHit), all trained on a unified 24-dimensional covariate vector combining six clinical variables (age, sex, years of education, MMSE score, ADAS-13 score, and APOE-ε4 status) with the 18 MRI-derived time-based descriptors, evaluated under a leakage-free stratified 5-fold cross-validation protocol on an analytical cohort of 661 MCI patients from ADNI. Third, a rigorous comparative analysis is provided that quantifies the discriminative performance of each model family, identifies the most informative neuroimaging descriptors within the Cox PH framework, and establishes a reproducible methodological benchmark for time-to-event prediction in Alzheimer’s disease.

## 2. Theoretical Framework

### 2.1. Fundamentals of Survival Analysis

Survival analysis is a branch of statistics designed to model the time elapsed until a specific event of interest occurs. Unlike classical regression, which predicts a continuous outcome, or classification, which predicts a binary label, survival analysis models a duration: how long until something happens. In this study, the event of interest is the conversion from MCI to Alzheimer’s disease, and the outcome for each patient is the pair $\left(T_{i},\delta_{i}\right)$, where $T_{i}$ is the observed follow-up time (months) and $\delta_{i}\in \left\{0,1\right\}$ indicates whether conversion was observed ($\delta_{i}=1$) or the patient was censored ($\delta_{i}=0$).

Censoring is a fundamental feature of longitudinal clinical data. A patient is right-censored when their observation ends before the event occurs, for example because the study period ends, the patient is lost to follow-up, or they withdraw consent. For such a patient, we do not know when (or whether) conversion will eventually occur; we know only that it had not occurred by time $C_{i}$. This partial information is valuable and must not be discarded. Standard classification or regression methods treat censored patients as non-events, introducing systematic bias. Survival models incorporate censoring correctly by conditioning every calculation on the information available up to $C_{i}$ [4].

Three mathematical functions characterize the distribution of survival times. The survival function S(t)=P(T>t) gives the probability that a subject remains event-free beyond time *t*; it is a monotonically decreasing function from S(0)=1 to S(∞)=0. The hazard function:

(1) $$h\left(t\right)=\lim_{\Delta t\to 0}\frac{P(t\leq T<t+\Delta t|T\geq t)}{\Delta t}$$

represents the instantaneous rate of the event at time *t* given that the subject has survived up to that point, the momentary risk of conversion. The cumulative hazard $H\left(t\right)=\int_{0}^{t}h\left(u\right)\;du$ accumulates this risk over time and is related to the survival function by S(t)=exp(−H(t)).

The Kaplan–Meier (KM) estimator [13] is the standard non-parametric tool for estimating S(t) without assuming any functional form. At each observed event time $t_{j}$ it multiplies survival probabilities:

(2) $$\hat{S}\left(t\right)=\prod_{t_{j}\leq t}\left(1-\frac{d_{j}}{n_{j}}\right)$$

where $d_{j}$ is the number of events and $n_{j}$ the number at risk at $t_{j}$. Censored subjects contribute to the at-risk set $n_{j}$ up to their censoring time and are correctly excluded afterward. The KM estimator is widely used for descriptive analyses stratified by clinical variables such as APOE4 genotype, sex, or age group.

The primary discriminative metric throughout this benchmark is the concordance index (C-index) [14]:

(3) $$C\text{-}\mathrm{index}=P({\hat{r}}_{i}>{\hat{r}}_{j}|T_{i}<T_{j},\;\delta_{i}=1)$$

which measures the probability that the model assigns higher predicted risk to the subject who converts first, among all comparable pairs where the earlier event is observed. A C-index of 0.5 corresponds to random prediction; 1.0 indicates perfect discrimination.

### 2.2. Classical Parametric and Semi-Parametric Models

Classical survival models were designed to estimate the relationship between clinical covariates and survival time while correctly handling censored observations through likelihood-based inference. Three models of this family are included in the benchmark, presented in order of decreasing parametric flexibility: Cox Proportional Hazards (CPH), which is semi-parametric (the baseline hazard $h_{0}\left(t\right)$ is left unspecified and estimated non-parametrically from data); Weibull Accelerated Failure Time (AFT), which is fully parametric (it commits to a Weibull-shaped baseline hazard); and Exponential regression, the most restrictive parametric case (it assumes a constant hazard over time). These three models are therefore not the same: they differ fundamentally in the assumptions they impose on $h_{0}\left(t\right)$, which affects both their flexibility and their interpretability under different data-generating processes.

**Cox Proportional Hazards (CPH) model:** The Cox model [5] is the most widely used regression model in clinical survival analysis. The core idea is to model the hazard of each patient as a product of two components: a baseline hazard $h_{0}\left(t\right)$ shared by all patients, which captures how risk evolves over time in the population, and a covariate-dependent multiplier that scales this baseline according to the patient’s covariates:

(4) $$h\left(t|z_{i}\right)=h_{0}\left(t\right)\;\exp\left(\beta^{⊤}z_{i}\right)$$

The model is semi-parametric: $h_{0}\left(t\right)$ is left completely unspecified and estimated non-parametrically via the Breslow estimator, while the covariate effects β are estimated by partial maximum likelihood. Critically, this partial likelihood discards $h_{0}\left(t\right)$ entirely and conditions only on the ordering of event times, allowing parameter estimation even without knowing the shape of the baseline risk. Censored patients contribute to the risk sets in the denominator up to their censoring time. Each coefficient $\beta_{k}$ is interpreted through its hazard ratio ${\mathrm{HR}}_{k}=\exp\left(\beta_{k}\right)$: a unit increase in covariate *k* multiplies the instantaneous conversion risk by ${\mathrm{HR}}_{k}$. The proportional hazards assumption, that this ratio remains constant over time, is the key structural constraint of the model.

**Weibull regression:** While Cox leaves $h_{0}\left(t\right)$ unspecified, parametric models commit to a specific functional form for the baseline hazard. The Weibull model [6] specifies:

(5) $$h\left(t|z_{i}\right)=\lambda \rho \;t^{\rho -1}\exp\left(\beta^{⊤}z_{i}\right)$$

where λ>0 is a scale parameter and ρ>0 is a shape parameter that controls how risk evolves over time. When ρ>1, the hazard increases with time, biologically consistent with neurodegeneration, where accumulated brain atrophy accelerates the instantaneous risk of conversion. When ρ<1 risk decreases over time; when ρ=1 the Weibull reduces to the Exponential model. All parameters are jointly estimated by full maximum likelihood, with censored observations contributing the term $P(T>C_{i})$ to the likelihood. Parametric specification enables closed-form survival probability predictions and model selection via the Akaike Information Criterion (AIC) and the Bayesian Information Criterion (BIC), which penalise model log-likelihood against parameter count to compare nested parametric families without requiring a likelihood-ratio test.

**Exponential regression:** The Exponential model is the simplest parametric model, obtained by fixing ρ=1 in the Weibull:

(6) $$h\left(t|z_{i}\right)=\lambda \;\exp\left(\beta^{⊤}z_{i}\right)$$

This implies a constant hazard over time: the instantaneous risk of conversion is the same at any point during follow-up, given the same covariates. Although this assumption is rarely realistic in neurodegeneration, the Exponential model serves as an important lower-bound reference in the benchmark, establishing the minimum performance achievable under the strongest parametric constraints.

### 2.3. Machine Learning for Survival Analysis

Classical survival models assume that covariates affect the hazard through a linear combination $\beta^{⊤}z_{i}$. Machine learning methods relax this constraint by learning non-linear and interactive covariate–outcome relationships directly from data. Incorporating survival analysis into ML frameworks requires two essential adaptations: (1) the optimization criterion must correctly handle censored observations, and (2) the model output must yield a valid survival estimate, a cumulative hazard H(t) or survival function S(t), rather than a class label.

**Random Survival Forest (RSF):** Random Forest is an ensemble method that constructs many independent decision trees on bootstrap samples of the data and averages their predictions. Each tree partitions the covariate space into regions (nodes) of increasingly homogeneous outcomes. To extend this to survival analysis, RSF [9] makes two fundamental changes.

First, the splitting criterion at each node is replaced by the log-rank statistic, which directly measures whether splitting the subjects in a node into two groups produces significantly different survival curves. This criterion naturally handles censoring: subjects who are censored contribute to the at-risk denominator in the log-rank test up to their censoring time and are then correctly removed, exactly as in the KM estimator.

Second, instead of a class label, each terminal node stores a Nelson–Aalen cumulative hazard estimate computed from the subjects that fall into that node. For a new patient, the tree routes them to a terminal node and returns that node’s cumulative hazard estimate. The final ensemble prediction averages across all *B* trees:

(7) $$\hat{H}\left(t|z_{i}\right)=\frac{1}{B}\sum_{b=1}^{B}{\hat{H}}_{b}\left(t|z_{i}\right)$$

RSF requires no parametric assumptions, handles non-proportional hazards and high-dimensional interactions naturally, and provides variable importance measures through permutation of out-of-bag predictions.

**Gradient Boosting Survival Trees (GBST):** Gradient boosting constructs an additive ensemble of weak learners (shallow trees) by fitting each new tree to the residual errors of the current ensemble. Unlike RSF, which builds all trees independently in parallel, GBST is a sequential algorithm: each new tree corrects the mistakes of the previous ones.

To adapt gradient boosting to survival analysis, GBST [10] defines the loss function as the negative Cox partial log-likelihood:

(8) $$L=-\;\sum_{i:\;\delta_{i}=1}\;\left[F\left(z_{i}\right)-\log\;\sum_{j\in R(t_{i})}\exp\left(F(z_{j})\right)\right]$$

where $F\left(z\right)=\sum_{m}\eta \;f_{m}\left(z\right)$ is the current ensemble prediction (a risk score analogous to $\beta^{⊤}z_{i}$ in Cox), η is the learning rate, and $R\left(t_{i}\right)=\left\{j:T_{j}\geq t_{i}\right\}$ is the risk set at each event time, which naturally excludes subjects censored before $t_{i}$. Only uncensored patients ($\delta_{i}=1$) appear in the outer sum; the inner summation over the risk set $R(t_{i})$, which appears inside the logarithm, plays the role of the normalising denominator of the Cox partial likelihood, accumulating the predicted risk of every patient still event-free at each observed event time. Each new tree $f_{m}$ is fitted to the negative gradient of L, corresponding to the difference between observed events and model-predicted risk accumulated over the risk sets. This sequential error correction gives GBST strong discriminative performance in moderate-dimensional settings and implicit feature selection through learning-rate regularization.

### 2.4. Deep Learning for Survival Analysis

Deep learning models go further than tree ensembles by learning hierarchical representations of input covariates through multiple layers of non-linear transformations. The central challenge is constructing a loss function that (1) correctly propagates gradients through the network and (2) handles censored observations without discarding their information. Two architectures are evaluated: DeepSurv, which extends the Cox partial likelihood with a neural network predictor, and DeepHit, which reformulates survival as a discrete-time classification problem eliminating the proportional hazards assumption entirely.

**DeepSurv:** The Cox model is limited by its linear predictor: the log-hazard is constrained to be a linear combination of covariates. DeepSurv [11] retains the Cox proportional hazards structure but replaces the linear predictor with a deep neural network $\phi (z_{i};\;\theta )$:

(9) $$h\left(t|z_{i}\right)=h_{0}\left(t\right)\;\exp\;\left(\phi (z_{i};\theta )\right)$$

The network ϕ(·) is a fully connected multi-layer perceptron with ReLU activations, batch normalization, and dropout. It maps the covariate vector $z_{i}$ to a single scalar risk score, the log relative hazard, learning complex non-linear interactions that a linear Cox model cannot capture. The network is trained end-to-end by minimizing the same negative Cox partial log-likelihood used in the classical Cox model. Censoring is handled identically: only uncensored patients contribute to the numerator (their observed event), while all patients still at risk, including censored ones, contribute to the denominator at each event time. After training, the baseline hazard $h_{0}\left(t\right)$ is estimated non-parametrically via the Breslow estimator, recovering a full survival function. DeepSurv thus inherits the interpretability of the Cox framework while gaining the flexibility of deep feature learning.

**DeepHit:** DeepSurv, like Cox, still requires proportional hazards: the relative risk between two patients must remain constant over time. DeepHit [12] eliminates this assumption entirely by reformulating survival prediction as a discrete-time classification problem.

The continuous follow-up period $\left[0,T_{\max}\right]$ is divided into *K* quantile-based intervals. Instead of predicting a risk score, the network directly estimates the probability that the event occurs in each specific interval:

(10) $$\hat{f}\left(t_{k}|z_{i}\right)=P\left(T=t_{k}|z_{i}\right),\;k=1,\ldots ,K$$

with the constraint $\sum_{k=1}^{K}\hat{f}\left(t_{k}\right)\leq 1$. The survival function is then recovered as:

(11) $$\hat{S}\left(t_{k}|z_{i}\right)=1-\sum_{j=1}^{k}\hat{f}\left(t_{j}|z_{i}\right)$$

The loss function combines two terms. The log-likelihood term accounts for censoring: for an observed event at $t_{k}$ it maximizes $\hat{f}\left(t_{k}\right)$; for a censored patient at $C_{i}$ it conditions on survival past $C_{i}$, maximizing $\hat{S}\left(C_{i}\right)$. The ranking loss directly penalizes concordance violations between pairs of subjects: if patient *i* converts before patient *j*, the model should assign higher risk to *i*:

(12) $$L_{\mathrm{DeepHit}}=L_{\log}+\alpha \;L_{\mathrm{rank}}$$

This formulation makes no assumption about the shape of risk over time and can capture non-proportional, non-monotone dynamics. Its main limitation is that reliable estimation requires sufficient events per time bin: with sparse data spread across many intervals, probability estimates become unstable.

### 2.5. Temporal Signal Representations for Neuroimaging

A distinctive contribution of this work is treating serial MRI brain volumes not as repeated scalar measurements but as discrete biomedical signals encoding the temporal dynamics of neurodegeneration. The validity of this encoding rests on two properties. First, each descriptor targets a statistically distinct aspect of the longitudinal trajectory: rate of change, trajectory roughness, and cumulative volume burden, which are well-established estimators in longitudinal biomarker analysis [15]. Second, the strict anti-leakage constraint (computing every descriptor exclusively from measurements prior to the event or censoring time, $t_{ij}\leq T_{i}$) preserves the causal temporal ordering required for valid survival prediction. Together, these properties guarantee that the extracted features reflect true biological progression rather than artifacts of observation schedule or outcome knowledge. This perspective enables the extraction of structured temporal features that capture trajectory information beyond what any single baseline measurement represents. These features are incorporated as covariates into all survival models, combined with six clinical variables to form a 24-dimensional covariate vector $z_{i}\in R^{24}$. Three scalar statistical descriptors are extracted per brain region per patient, where r∈{1,…,6} indexes the anatomical region, each summarizing a distinct aspect of the volumetric trajectory. The atrophy rate (slope ${\hat{\beta }}_{1}^{\left(r\right)}$) is estimated via ordinary least squares fitted to the ICV-normalized volume series of region *r*, providing the average rate of volumetric change per month. A negative slope in cortical regions (hippocampus, entorhinal cortex) reflects progressive atrophy consistent with neurodegeneration; a positive slope in ventricular volume reflects expansion accompanying neuronal loss. The variability $\sigma^{\left(r\right)}$ is the population standard deviation of normalized volumes around their visit-wise mean, capturing intra-subject fluctuation across the observation window. The time-weighted cumulative mean $\mu^{\left(r\right)}$ integrates the volumetric trajectory via the trapezoid rule, normalized by total follow-up duration to prevent implicit encoding of observation length. Together, these three descriptors per region yield 18 neuroimaging covariates directly interpretable in terms of established AD neurobiology.

All three descriptors per region are computed exclusively from measurements occurring before the event or censoring time ($t_{ij}\leq T_{i}$), enforcing strict temporal anti-leakage. The resulting 18 neuroimaging descriptors are concatenated with the six clinical baseline covariates to form the augmented covariate vector $z_{i}\in R^{24}$ used across all seven survival models evaluated in this benchmark. This representation is computationally efficient, requires no architectural modifications to any model, and is biologically interpretable: negative cortical slopes reflect progressive atrophy; positive ventricular slopes reflect neuronal loss-associated expansion.

## 3. Materials and Methods

### 3.1. Dataset and Cohort Construction

Data were obtained from the ADNI database (https://adni.loni.usc.edu, accessed on 15 January 2025) [8], a multi-site longitudinal study collecting clinical, neuroimaging, and genetic data across the AD spectrum. All participants classified as MCI (LMCI or EMCI) at baseline were included under the following criteria: (i) age 50–95 years; (ii) follow-up 1–180 months; (iii) available APOE4 genotype; and (iv) at least three serial MRI observations per brain region to enable temporal feature extraction.

The primary outcome was conversion to AD, defined as a change in diagnostic classification at any subsequent visit. Participants who did not convert were right-censored. Applying all inclusion criteria yielded an analytical cohort of 661 MCI patients with 250 conversion events (event rate 37.8%, median follow-up 42.0 months).

For each subject *i*, the data tuple is:

(13) $$D={\left\{\left(T_{i},\;\delta_{i},\;z_{i}\right)\right\}}_{i=1}^{N}$$

where $T_{i}$ is observed follow-up time (months), $\delta_{i}\in \left\{0,1\right\}$ is the event indicator, and $z_{i}\in R^{24}$ is the augmented covariate vector comprising six clinical variables and 18 neuroimaging temporal descriptors. Events per variable: EPV = 250/24 ≈ 10.4.

### 3.2. Missing Data Handling

Patients with missing APOE4 genotype were excluded because imputation would alter genetic risk stratification. Patients lacking complete neuroimaging series (≥3 observations per brain region) were excluded, as temporal descriptors cannot be reliably estimated from fewer observations. The threshold of ≥3 MRI sessions in ADNI was chosen to enable stable OLS estimation of the atrophy slope ${\hat{\beta }}_{1}^{\left(r\right)}$, which requires at least three time-points to yield a residual degree of freedom. In OASIS-3, the threshold was lowered to ≥2 sessions because the available pool of subjects meeting the ADNI criterion (≥3 sessions) was insufficient for a five-fold stratified cross-validation analysis with adequate events per fold; the slope was still estimated but with higher uncertainty, which is acknowledged as an additional limitation of the OASIS-3 analysis. This criterion difference between the two cohorts is discussed in Section 5. As a sensitivity analysis, we applied the ≥2 session threshold to the ADNI cohort; this yielded a larger analytical cohort (n=824, 316 events, 38.3%) and a Cox PH C-index of 0.858±0.030, compared with 0.863±0.027 in the main analysis (n=661), a negligible difference of Δ=−0.005. The minimal performance change confirms that the main results are robust to this criterion and that the ≥3 session threshold provides marginally more stable descriptor estimation. Missing MMSE and ADAS13 values at baseline (<2% of records) were handled by listwise deletion. No multiple imputation was performed to avoid introducing uncertainty into survival outcomes.

### 3.3. Clinical Covariates

Six baseline variables were selected based on established predictive value for MCI-to-AD conversion [2,16]:

(14) $$x_{i}={\left[{\mathrm{AGE}}_{i},\;{\mathrm{SEX}}_{i},\;{\mathrm{EDU}}_{i},\;\mathrm{APOE}4_{i},\;{\mathrm{MMSE}}_{i},\;\mathrm{ADAS}13_{i}\right]}^{⊤}$$

**Age (AGE):** Age at baseline (range 54–91 years, mean 72.7±7.5). Controls for biological aging effects. **Sex (SEX):** Binary (1 = male, 0 = female); 60% male. **Education (EDU):** Years of formal education (range 4–20, mean 16.0±2.8); proxy for cognitive reserve. **APOE4 Genotype:** Number of ε4 alleles (0, 1, or 2); strongest known genetic risk factor for late-onset AD [17]: 51.4% non-carriers, 38.0% heterozygous, 10.6% homozygous. **MMSE:** Mini-Mental State Examination (0–30) [18]; mean 26.5±3.7. **ADAS13:** 13-item cognitive battery (0–85; higher = worse) [19]; mean 18.4±10.6.

### 3.4. Neuroimaging Biomarkers and ICV Normalization

Volumetric measurements were derived via FreeSurfer automated segmentation (v5.1+) [20] for six brain regions: (1) Hippocampus, (2) Entorhinal cortex, (3) Fusiform gyrus, (4) Middle temporal cortex, (5) Lateral ventricles, and (6) Whole brain. These regions are well-validated markers of AD-related neuroanatomical progression [2].

All volumes were normalized by estimated intracranial volume (ICV) [21]:

(15) $${\tilde{x}}_{ij}^{\left(r\right)}=\frac{x_{ij}^{\left(r\right)}}{{\mathrm{ICV}}_{i}}\times 1000$$

where $x_{ij}^{\left(r\right)}$ is the raw volume of region *r* for subject *i* at visit *j*. The resulting ICV-normalized measurements were organized as serial biomedical signals $s_{i}^{\left(r\right)}={\left\{\left(t_{ij},\;{\tilde{x}}_{ij}^{\left(r\right)}\right)\right\}}_{j=1}^{n_{i}}$, with temporal truncation $t_{ij}\leq T_{i}$ enforced to prevent information leakage.

### 3.5. Temporal Feature Extraction

#### Statistical Descriptors

Three complementary scalar descriptors were extracted per signal (18 total across 6 regions):

**Rate of atrophy (slope):** From ordinary least squares ${\tilde{x}}^{\left(r\right)}\left(t\right)\approx \beta_{0}^{\left(r\right)}+\beta_{1}^{\left(r\right)}t$, where ${\mathrm{slope}}_{i}^{\left(r\right)}={\hat{\beta }}_{1}^{\left(r\right)}$.

**Variability (σ):** Population standard deviation of ICV-normalized volumes around their visit-wise mean:

(16) $$\sigma_{i}^{\left(r\right)}=\sqrt{\frac{1}{n_{i}}\sum_{j=1}^{n_{i}}\;{\left({\tilde{x}}_{ij}^{\left(r\right)}-{\bar{\tilde{x}}}_{i}^{\left(r\right)}\right)}^{2}}$$

where ${\bar{\tilde{x}}}_{i}^{\left(r\right)}=\frac{1}{n_{i}}\sum_{j=1}^{n_{i}}{\tilde{x}}_{ij}^{\left(r\right)}$ is the visit-wise mean. This descriptor quantifies intra-subject amplitude variability across the observation window, independently of the atrophy trend captured by ${\hat{\beta }}_{1}^{\left(r\right)}$.

**Time-weighted mean (μ):**

(17) $$\mu_{i}^{\left(r\right)}=\frac{1}{t_{i,n_{i}}-t_{i,1}}\sum_{j=1}^{n_{i}-1}\frac{{\tilde{x}}_{ij}^{\left(r\right)}+{\tilde{x}}_{i,j+1}^{\left(r\right)}}{2}\;\left(t_{i,j+1}-t_{ij}\right)$$

Normalizing by follow-up duration prevents implicit encoding of observation length. The 18 descriptors are concatenated with the 6 clinical variables to form the augmented covariate vector $z_{i}\in R^{24}$ (events per variable EPV =250/24≈10.4, consistent with the EPV reported in Section 3.1).

### 3.6. Survival Models

The seven survival models evaluated in this benchmark (Cox PH, Weibull AFT, Exponential, RSF, GBST, DeepSurv, and DeepHit) are described mathematically in Section 2 (Equations (4)–(12)). All models received the same augmented covariate vector $z_{i}\in R^{24}$ as input. L2 regularization (α=0.1) was applied to Cox PH and Weibull AFT to stabilize estimation under moderate EPV (≈10.4). Complete hyperparameter configurations are consolidated in Table 1.

### 3.7. Model Training and Hyperparameter Configuration

No formal hyperparameter search was conducted; all values were set to principled defaults from each model’s original publication and are reported in full for reproducibility. Table 1 consolidates the key settings for all models. It should be noted that DeepSurv was trained for a fixed 100 epochs (the default in the original publication [11]), whereas DeepHit used early stopping with patience 20, up to 300 epochs. This asymmetry is a deliberate design choice of this benchmark: all models were configured according to their published reference implementations to ensure reproducibility against the existing literature. The training protocol asymmetry constitutes a limitation of the comparison between these two architectures, as discussed in Section 5.

### 3.8. Software and Reproducibility

All analyses used Python 3.12. Classical models: lifelines 0.30. Ensemble models: scikit-survival 0.27. Neural models: pycox 0.3 with torchtuples and PyTorch 2.1. Data processing: pandas 2.0 and numpy 1.26. All random seeds fixed to 42 for reproducibility. Analysis code is openly available at https://github.com/NathaliaArias/adni-survival-benchmark (accessed on 15 January 2025) (deposited under MIT licence); a Zenodo archive with a citable DOI will be registered upon acceptance.

### 3.9. Evaluation Protocol

All seven models were evaluated on the analytical cohort (n=661) using the augmented covariate vector $z_{i}\in R^{24}$ under stratified 5-fold cross-validation (random seed = 42), with event status as the stratification variable. Three strict anti-leakage measures were enforced: (1) temporal truncation ($t_{ij}\leq T_{i}$) before any feature computation, ensuring no post-event observations contaminate the descriptors; (2) per-fold standardization of continuous covariates fitted exclusively on training-fold subjects and applied to the held-out fold; (3) per-fold estimation of Cox baseline hazards on training data only.

The primary metric was the concordance index (C-index) [14]:

(18) $$C\text{-}\mathrm{index}=P({\hat{r}}_{i}>{\hat{r}}_{j}|T_{i}<T_{j},\;\delta_{i}=1)$$

reported as mean ± standard deviation across five folds. A C-index of 0.5 indicates random discrimination; 1.0 indicates perfect concordance. Multicollinearity among the 24 covariates was assessed via the Variance Inflation Factor (VIF), with threshold <5 [6].

## 4. Results

### 4.1. Cohort Characteristics

Cohort characteristics are summarized in Table 2. The primary cohort (993 patients, 376 events) was filtered to the analytical cohort by requiring at least three serial MRI observations per brain region; the two cohorts are demographically similar, confirming that the neuroimaging constraint did not introduce selection bias with respect to the primary conversion outcome, although some differences were observed and residual selection bias cannot be excluded. Of the 993 primary cohort patients, 332 (33.4%) were excluded by the ≥3 MRI session criterion. A formal comparison of all baseline characteristics between included (n=661) and excluded (n=332) participants is presented in Table 3. The two groups had an identical conversion rate (37.8% vs. 38.0%, p=1.000), similar sex distribution (58.7% vs. 60.5% male, p=0.625), and indistinguishable APOE4 burden across all three genotype categories (p>0.59). However, excluded patients were slightly older (74.5±7.5 vs. 72.2±7.4 years, p<0.001), had marginally lower MMSE (27.3±1.9 vs. 27.7±1.8, p=0.002), higher ADAS-13 (17.9±6.9 vs. 15.7±6.6, p<0.001), and a substantially shorter median follow-up (24.5±20.2 vs. 54.4±37.9 months, p<0.001), consistent with early withdrawal from the serial imaging protocol. The absence of a difference in conversion rate, sex, and APOE4 distribution suggests that the ≥3 MRI session criterion did not introduce major selection bias with respect to the primary outcome; nevertheless, the observed differences in age, cognitive scores, and follow-up duration indicate that residual selection bias cannot be fully excluded.

Kaplan–Meier analysis confirmed a dose–response gradient by APOE4 genotype (log-rank p<0.001), with homozygous carriers (ε4/ε4) showing the shortest time to conversion, consistent with established genetic risk patterns [17].

### 4.2. Ablation Analysis: Incremental Contribution of Temporal MRI Descriptors

To quantify the incremental contribution of the temporal MRI descriptors, a feature-set ablation was conducted using Cox PH (the most interpretable model in the benchmark) on three nested input configurations: (A1) six clinical variables only; (A2) six clinical variables plus six ICV-normalised first-visit MRI volumes (one per brain region; baseline snapshot, no trajectory information; 12 variables total); and (A3) the full 24-dimensional covariate vector replacing the six baseline volumes with 18 temporal descriptors (slope, σ, μ per region). All three configurations were evaluated under identical 5-fold stratified cross-validation. Results are presented in Table 4.

The clinical-only model (A1) achieved a C-index of 0.794±0.035, confirming that age, sex, education, APOE4 genotype, MMSE, and ADAS-13 alone provide meaningful but limited prognostic discrimination. Adding a single baseline MRI snapshot (A2) improved performance to 0.814±0.022 (Δ=+0.021), demonstrating that volumetric information from a single visit contributes independent prognostic signal beyond clinical variables. Replacing the baseline MRI volumes with temporal descriptors (A3) yielded a further gain to 0.863±0.027 (Δ=+0.049 over A2; Δ=+0.070 over A1), demonstrating that longitudinal trajectory information (rate of atrophy, intra-subject variability, and time-weighted cumulative mean) provides substantial additional discriminative value beyond what a single baseline scan can capture. These results confirm that the performance gain of the full pipeline is attributable to the temporal structure of the longitudinal MRI data rather than merely to the inclusion of neuroimaging features.

### 4.3. Survival Model Benchmark

Table 5 presents the 5-fold cross-validation C-index and Integrated Brier Score (IBS) for all seven survival models, all trained on the 24-dimensional covariate vector (6 clinical + 18 temporal MRI descriptors).

All seven models converged to a narrow C-index band of 0.853–0.863 (ΔC-index =0.010). Four models tied at the top of the ranking to three decimal places: DeepHit (0.863±0.017), Cox PH (0.863±0.027), Weibull AFT (0.863±0.026), and Exponential (0.863±0.026). That a single-parameter parametric model (Exponential) matches a deep neural network (DeepHit) is not surprising in this setting: once the longitudinal MRI trajectories are compressed into 24 structured covariates, all model families access essentially the same discriminative information, and the no clear performance separation is observed among the evaluated model architectures, which may be consistent with feature-level constraints, though the cause cannot be established from these results alone. DeepHit achieves the lowest fold-to-fold variability (±0.017), suggesting more stable gradient-based optimization under this feature regime. This convergence suggests that, within this specific feature-engineering paradigm and for the 24-dimensional hand-engineered representation used here, discriminative performance appears to be bounded by feature quality rather than model architecture—though this conclusion should not be generalised beyond the current experimental setting: once the temporal MRI trajectories are encoded as structured covariates, no model family extracted meaningfully additional signal in this benchmark. Calibration results (IBS, Table 5) confirm good predictive accuracy for all evaluated models: Cox PH (0.084) and GBST (0.089) achieve the best calibration, all substantially below the null-model threshold of 0.25. Figure 1 summarises the C-index distribution across models.

To formally assess whether the observed ranking differences are statistically meaningful, pairwise Wilcoxon signed-rank tests were applied to the five per-fold C-index vectors of all 21 model pairs. All comparisons were non-significant (p≥0.125 for all pairs), consistent with the narrow ΔC-index =0.010 band. This result should be interpreted cautiously: with only five paired observations (folds), the test has low statistical power and cannot distinguish equivalence from insufficient evidence. The non-significant results confirm that no model *dominates* the others at this sample size; model selection should therefore be guided by interpretability, computational requirements, and deployment constraints rather than by C-index alone.

### 4.4. Time-Dependent Discriminative Performance

The global C-index characterises overall ranking ability across the entire follow-up horizon; clinical decision-making additionally requires discrimination to be evaluated at concrete prognostic windows. Table 6 reports the time-dependent AUC [22] at 12, 24, 36, and 48 months for the three representative models (Cox PH, RSF, GBST), computed via 5-fold stratified CV on the full 24-variable feature set.

All three models sustain AUC well above the random baseline (0.5) across all four clinical windows, with performance increasing at longer horizons. GBST achieves the highest early-window AUC (0.891 at 12 months), while Cox PH reaches 0.938 at 48 months. The upward trend reflects the cumulative nature of the dynamic AUC estimator as the pool of observed events grows; importantly, AUC > 0.85 at 12 months indicates clinically useful discrimination even at early prognostic windows. Figure 2 illustrates the AUC trajectories for the three models with 95% confidence bands.

Risk stratification was further assessed by dividing the cohort into quartiles according to the Cox PH log-partial-hazard score and plotting Kaplan-Meier survival curves for each group (Figure 3). A highly significant log-rank test (p<0.001) confirms that the model stratifies patients into clinically meaningful risk tiers: the high-risk quartile (Q4) converts to dementia at substantially earlier times than the low-risk quartile (Q1), providing direct evidence of clinical utility beyond point discrimination metrics.

### 4.5. Significant Predictors in the Cox PH Model

Table 7 reports hazard ratios for the 11 covariates significant at p<0.05 in the Cox PH model fitted on the 24-dimensional vector (n=661, L2 penalty α=0.1). Thirteen covariates were non-significant and are omitted for brevity.

Ventricular expansion slope (HR = 1.42) and ADAS13 (HR = 1.62) were the strongest risk amplifiers. All six cortical slopes showed protective HRs < 1, consistent with the established neuroanatomical pattern of MCI-to-AD conversion: patients who retain greater cortical volume over time have lower conversion risk [2]. APOE4 genotype remained a significant clinical predictor (HR = 1.21) after adjusting for all 18 MRI descriptors, confirming its independent prognostic contribution. Age, sex, and education were not significant after controlling for cognitive scores and imaging biomarkers.

Multicollinearity was assessed via the Variance Inflation Factor (VIF). All 24 covariates yielded VIF <5 (Table 8), with a maximum of 4.81 ($\sigma_{\mathrm{WholeBrain}}$) and a median of 2.33. The five highest VIF values correspond to $\sigma^{\left(r\right)}$ and $\mu^{\left(r\right)}$ descriptors, which is expected because $\sigma^{\left(r\right)}$ captures inter-visit variability correlated across anatomically proximal regions. All VIF values remain well below the moderate threshold of 5, confirming that the 24-covariate model is statistically valid with no redundant predictors [6].

### 4.6. Variable Importance: Random Survival Forest and Gradient Boosting

Figure 4 shows permutation-based variable importance (RSF) and gradient-boosted feature importance (GBST) for the 24 covariates. In both ensemble methods, ADAS13 and the ventricular expansion slope emerge as the two dominant predictors, consistent with the Cox PH hazard ratios (Table 7). Temporal MRI descriptors collectively account for the majority of predictive importance, confirming that the atrophy-rate and variability features extracted from serial volumetrics capture prognostic information beyond static clinical scores.

### 4.7. Exploratory Analysis on an Independent OASIS-3 Cohort

An exploratory cross-cohort analysis of the temporal descriptor pipeline was conducted on the OASIS-3 cohort [23]. From 310 subjects with baseline CDR =0.5 (MCI), 88 had at least two valid FreeSurfer sessions (QC status: Passed or Passed with edits) with complete volumetric data after temporal truncation (the same anti-leakage constraint applied in ADNI). After merging with clinical records and removing subjects with missing APOE4 or MMSE, the OASIS-3 analytical cohort comprised n=78 subjects with an event rate of 35.9% (28 conversions; CDR total ≥1) and a median follow-up of 96.6 months. The conversion outcome in OASIS-3 (CDR total ≥1, corresponding to mild or greater dementia) is clinically comparable to the ADNI outcome (change in diagnostic classification to dementia): a CDR total score of 1 represents mild dementia consistent with an Alzheimer’s dementia diagnosis [24], and this correspondence has been used in prior cross-cohort survival analyses combining ADNI and OASIS [25]. Residual heterogeneity between the two conversion criteria remains a limitation and is discussed in Section 5. Because ADAS-13 is not collected under the OASIS-3 UDS protocol, the external model used 23 variables (5 clinical: AGE, SEX, EDUC, APOE4, MMSE; 18 temporal MRI descriptors), and the Exponential AFT model was excluded due to an API incompatibility in lifelines (v0.30); its ADNI performance was identical to Weibull AFT (0.863±0.026).

Table 9 reports the 5-fold CV C-index for each of the six evaluated models, together with the corresponding ADNI results for direct comparison.

These results are exploratory given the small per-fold sample size in OASIS-3 (EPV ≈ 1 per training fold, far below the Peduzzi convention of ≥10); they are reported for hypothesis generation and should not be interpreted as confirmatory external validation. With this caveat, a preliminary stratification by model family is observed (Table 9). Classical models yielded C-index 0.739–0.760 with moderate fold-to-fold variability, and RSF (0.759±0.049) matched Weibull AFT (0.760±0.063) despite originating from an entirely different modelling paradigm. The preliminary mean drop relative to ADNI was 0.094–0.124 C-index points [2,23]; as noted in Section 4.7, this drop is additionally confounded by the absence of ADAS-13 from OASIS-3.

In contrast, deep learning and ensemble models degraded substantially. DeepSurv fell to 0.551±0.116 (Δ=−0.308), near the random-classifier baseline. GBST dropped to 0.656±0.107 (Δ=−0.206), and DeepHit to 0.651±0.240 (Δ=−0.212). The high variability of DeepHit (±0.240) represents a sharp reversal from its ADNI performance (±0.017), consistent with the inability of discrete-time networks to estimate stable bin probabilities under the approximately five events per fold available in OASIS-3.

These preliminary results are directionally consistent with the hypothesis that classical penalised models extrapolate more gracefully under covariate distribution shift and data-scarce conditions than higher-capacity architectures, a pattern documented in meta-analyses of survival benchmarks at comparable sample sizes [3]. Confirmatory validation on a larger cohort with full covariate parity is required before drawing confirmatory conclusions. The practical implication is that Cox PH and Weibull AFT, available in standard statistical software without GPU requirements, are the most stable formulations for research deployment of this pipeline under data-scarce conditions; confirmatory prospective validation would be required before clinical adoption.

As a complementary calibration metric, the Integrated Brier Score (IBS) of the Cox PH model in OASIS-3 was 0.179±0.050, evaluated at the 25th, 50th, and 75th percentiles of observed event times (55, 80, and 158 months). This represents a 28.3% improvement over the null-model IBS of 0.25 (Kaplan–Meier baseline), confirming that the predicted survival probabilities are meaningfully calibrated beyond the ranking measured by the C-index.

Figure 5 illustrates the per-model C-index comparison between both cohorts.

## 5. Discussion

A central contribution of this benchmark is the transformation of serial MRI volumetric trajectories into structured statistical descriptors (atrophy rate, variability, and time-weighted cumulative mean) that are directly consumable by any survival model family without architectural modifications. This approach is computationally efficient, interpretable within established AD neurobiology, and robust to irregular visit spacing characteristic of real-world clinical cohorts, aligning with prior temporal feature engineering work in AD [7] while extending it to a systematic comparison across seven model families.

The primary finding, that all seven models converge to a narrow C-index band (Δ=0.010), suggests a direct methodological interpretation: within this specific feature-engineering paradigm, when the input features already encode the relevant temporal dynamics of neurodegeneration, performance appears bounded by feature quality rather than model architecture—a conclusion limited to the 24-dimensional hand-engineered representation evaluated here. End-to-end architectures operating directly on raw volumetric sequences could recover signal beyond what three scalar descriptors per region capture, and constitute a natural direction for future investigation. Importantly, this benchmark characterises the behaviour of survival algorithms on a fixed 24-dimensional hand-engineered representation; it does not establish architecture-independence of the underlying prediction problem, and end-to-end temporal deep learning approaches operating on raw longitudinal sequences may potentially surpass the discriminative ceiling imposed by this feature set. Among the neural models, DeepHit achieved the top ranking with the lowest fold-to-fold variability, a stability advantage attributable to its discrete-time formulation (which relaxes the proportional hazards assumption) and its ranking-aware loss function that directly optimises concordance order across folds. Tree ensembles and neural networks gain discriminative advantage over classical models primarily when raw or high-dimensional inputs require non-linear feature discovery; here, the temporal descriptors perform that discovery explicitly and interpretably, leaving little margin for more complex architectures. This is consistent with published meta-analyses [3]: at sample sizes of a few hundred to low thousands with predominantly monotone covariate effects and moderate feature dimensionality, classical models match deep architectures in point performance.

An important caveat qualifies the comparison between deep learning and classical models. Neither DeepSurv nor DeepHit underwent hyperparameter optimisation beyond the reference defaults listed in Table 1: learning rate, network width, dropout rate, and, for DeepHit, the number of discrete time bins were all fixed to their published reference values. Deep learning survival models are known to be sensitive to these choices [3], and the C-indices reported for DeepSurv (0.859±0.025) and DeepHit (0.863±0.017) should therefore be treated as conservative estimates of achievable performance rather than as calibrated head-to-head benchmarks against classical models. Had a nested cross-validation with Bayesian hyperparameter search been applied, the deep learning models might have attained higher C-indices, potentially narrowing the performance gap between the unoptimised DL results and the reference classical models within the current feature regime. This choice was deliberate: a reproducibility-first benchmark with fixed published defaults allows direct comparison with prior reported results and avoids the optimism bias introduced by tuning on the same data. Nevertheless, the relevant implication for the convergence finding is not that deep learning is equivalent to Cox PH, but that the hand-engineered 24-dimensional descriptor space sets an upper bound on performance that no model family, optimised or not, is able to exceed substantially with the available data.

Exploratory analysis on OASIS-3 produced a secondary finding of comparable practical relevance, and the observed cross-cohort degradation pattern illustrates potential domain-shift challenges that would need to be addressed before any future clinical application. Classical models maintained robust discrimination with moderate and architecturally consistent drops, while deep learning and ensemble models degraded substantially: DeepSurv approached the random-classifier baseline, and DeepHit’s fold-to-fold stability (its principal ADNI advantage) inverted entirely in the smaller external cohort. The training dynamics reveal a difference between the two architectures: DeepSurv’s validation loss begins to diverge upward from the training loss after approximately epoch 40 and continues rising through epoch 100, indicating a mild overfitting regime in the source cohort. DeepHit’s validation loss, in contrast, remains stable throughout, with early stopping triggering between 93 and 135 epochs across folds. It should be noted, however, that this comparison is not fully controlled: DeepSurv was trained for a fixed 100 epochs without early stopping, while DeepHit used early stopping with patience 20 up to 300 epochs (see Table 1). This training protocol asymmetry is a confound that prevents attributing the cross-cohort gap (Δ=−0.308 for DeepSurv vs. Δ=−0.212 for DeepHit) purely to architectural differences. Retraining DeepSurv with early stopping under an otherwise identical protocol is required to isolate the effect of architecture from the effect of training duration, and is planned for future work.

Several complementary mechanisms explain this stratification. First, statistical capacity: neural networks require larger training sets to constrain their parameter spaces; with approximately 62 training subjects per OASIS-3 fold, the networks under-train and produce unstable risk scores, whereas penalised Cox PH and RSF are explicitly regularised for small-sample regimes. Second, scanner and protocol heterogeneity: ADNI enforces a standardised 3T multi-site acquisition protocol [2], whereas OASIS-3 aggregates scans across diverse field strengths and acquisition parameters at a single centre (Knight ADRC, Washington University). FreeSurfer segmentation accuracy is sensitive to field strength and acquisition sequence; even minor volumetric offsets between cohorts can shift the covariate distributions that neural networks, unlike regularised classical models, cannot accommodate without domain adaptation. Third, demographic distribution shift: ADNI applies selective enrolment criteria that limit comorbidities and demographic diversity, while OASIS-3 is community-recruited, resulting in age and clinical heterogeneity differences that affect the atrophy trajectory descriptors serving as model inputs. Collectively, these factors produce a covariate distribution mismatch that amplifies with model complexity: penalised linear models extrapolate more gracefully under distribution shift than non-linear architectures whose decision surfaces are shaped by the training distribution. Domain adaptation methods such as transfer learning, instance reweighting, or covariate normalisation across sites represent a direct avenue to narrow this performance gap in future work.

Based on these exploratory findings, should the temporal descriptor pipeline be further developed towards clinical use, Cox PH and Weibull AFT appear to be preferable formulations for settings with limited data, which is the norm outside large research consortia, offering interpretable hazard ratios, stable cross-cohort performance in this exploratory setting, and no GPU requirements. Prospective validation on independent cohorts with full covariate parity would be required before any clinical translation.

Among prior work using longitudinal structural MRI on ADNI for MCI-to-AD conversion prediction, Platero and Tobar [25] is methodologically closest: it applies RSF with serial MRI features on an ADNI MCI cohort, combining temporal neuroimaging trajectories with survival analysis in a design directly comparable to the present study. The temporal descriptor pipeline introduced here builds on this direction using simpler scalar descriptors (slope, σ, μ) applied across a larger analytical cohort (n=661) and evaluated under strict anti-leakage cross-validation. More recent work has reported higher RSF C-indices on larger ADNI MCI subcohorts. Jahani et al. [26] evaluated classical and machine-learning survival models on n=902 ADNI MCI subjects using 61 baseline features spanning neuropsychological assessments, structural MRI, and CSF biomarkers, finding that RSF achieved the highest C-index of 0.878 (95% CI: 0.877–0.879) and lowest IBS. Saeed et al. [27] applied RSF to distinguish early from late MCI conversion trajectories on ADNI, reporting C-indices of 0.90 for eMCI and 0.82 for lMCI using multimodal inputs. Song et al. [28] similarly demonstrated the advantage of RSF over Cox PH in predicting progression to clinical AD dementia in ADNI. Sarica et al. [29] further examined RSF explainability via SHAP on ADNI for MCI conversion risk, emphasising the interpretability gains of tree-based survival models. The higher C-indices in these studies are attributable primarily to richer input modalities—PET, CSF, and genetic biomarkers alongside structural MRI—rather than to model architecture differences. The present benchmark deliberately restricts input to routine structural MRI to assess how far clinical-grade serial volumetrics alone can drive performance; direct numerical comparison with multimodal pipelines is therefore not interpretable without covariate-matched study designs [3]. A broader numerical comparison against other published studies is not reported here, as heterogeneity in cohort composition, outcome definitions, input modalities, and evaluation protocols across the literature precludes interpretable direct benchmarking without a formal systematic review methodology [3].

**Clinical Implications.** The practical utility of a prognostic model in memory clinics depends not only on statistical discrimination but on whether risk scores translate into actionable patient triage. Time-dependent AUC exceeding 0.85 at the 12- and 24-month horizons (Table 6) indicates that once sufficient longitudinal MRI data have been accumulated, the pipeline can reliably identify patients likely to convert within the next two years, a window directly relevant to enrolment in disease-modifying trials and to patient counselling regarding anticipated care needs in a research context. The Kaplan-Meier stratification by risk quartile (Figure 3) further illustrates that a simple binary threshold, separating Q1+Q2 from Q3+Q4, would yield two clinically distinct patient groups with substantially different conversion trajectories (log-rank p<0.001). Crucially, the pipeline requires only routine T1-weighted MRI and six standard clinical variables, all obtainable within a diagnostic neuroimaging appointment, with no need for PET, CSF sampling, or specialised sequences. This favourable cost-to-information ratio suggests the temporal descriptor approach as a promising methodological starting point for research-oriented decision support in memory clinic settings where multimodal biomarker collection is impractical or unavailable; prospective clinical validation is required before operational deployment.

Eight limitations apply to this study. First, the pipeline constitutes a dynamic risk prediction framework conditioned on the full longitudinal MRI history available up to the time of assessment ($t_{ij}\leq T_{i}$), not a baseline predictor applicable at first clinical contact. Predictions cannot be generated at the initial MCI diagnosis; at least two to three serial MRI sessions are required before a stable risk estimate can be computed. Future work should formalise this as a landmark analysis with fixed prediction horizons (e.g., assessing risk from months 6, 12, 24 onward), in which descriptors are recomputed from data available up to each landmark time only. Second, the cross-cohort comparison between ADNI and OASIS-3 is partially confounded by a predictor asymmetry: ADAS-13 is not collected under the OASIS-3 UDS protocol, so the external model was necessarily fitted on 23 rather than 24 variables. Because ADAS-13 is the strongest predictor in the full ADNI model (HR = 1.616, p<0.001), its absence from OASIS-3 depresses the external C-index relative to what the complete pipeline would achieve under full covariate parity. The reported OASIS-3 values (0.739–0.760 for classical models) therefore represent a lower bound on cross-cohort performance rather than the true drop of the 24-variable pipeline; the observed deltas (Δ=0.094–0.124 for classical models) conflate genuine domain shift with this predictor gap and should not be interpreted as the full performance loss under covariate parity. Replication on the National Alzheimer’s Coordinating Center (NACC, n>40,000 UDS records), which collects ADAS-13 under the same UDS protocol as ADNI, will provide statistically stable estimates with full covariate parity and adequate EPV, while also enabling competing-risk modelling that jointly accounts for death and non-AD dementia. Third, the proportional hazards assumption of Cox PH was tested post-hoc via Schoenfeld residuals using a rank-time transformation [30]. Of 24 covariates, 22 satisfied the assumption (p>0.05); two trajectory-variability descriptors exhibited time-varying coefficients: Hippocampus_*σ*_ ($\chi^{2}=9.69$, p=0.002) and Ventricles_*σ*_ ($\chi^{2}=5.83$, p=0.016). Three considerations bound the practical impact of these violations. First, the C-index is a purely rank-based statistic and is therefore robust to proportional hazards violations; the reported benchmark C-indices are not affected by the non-proportionality of these two descriptors. Second, the violations are confined to trajectory-variability features (σ), not to the primary atrophy-rate (slope) features; the non-proportionality is clinically interpretable as reflecting the Weibull finding that conversion risk accelerates over time (shape parameter $\hat{\rho }>1$), an intrinsic characteristic of the disease trajectory rather than a model misspecification. Third, as an empirical sensitivity check, we fitted a stratified Cox PH model in which Hippocampus_*σ*_ and Ventricles_*σ*_ serve as stratification factors, giving each stratum its own baseline hazard and thereby lifting the proportionality constraint for those two variables. The stratified model yielded a C-index of 0.844±0.029 (Δ=−0.019 relative to the standard Cox PH, 0.863±0.027). Critically, this reduction reflects the exclusion of these two variables from the linear predictor—since stratified variables contribute to the stratum-specific baseline hazard but not to the ranking risk score on which the C-index depends—not a consequence of the proportional hazards violation itself. The drop is driven primarily by the removal of Ventricles_*σ*_ (HR =0.702, p<0.001, Table 7), which was one of the strongest predictors in the model; Hippocampus_*σ*_, although it violates proportionality, did not attain significance in the Cox model and contributes minimally to the C-index reduction. The Weibull AFT model, which imposes no proportionality constraint and retains all 24 covariates as continuous predictors in the linear predictor, achieved C-index 0.863±0.026, identical to the standard Cox PH. Together, these results confirm that the discriminative performance reported in Table 5 is not an artifact of the proportional hazards violation: the correct PH-free formulation (Weibull AFT) reproduces the same C-index exactly, while the stratified Cox drops only because it loses predictive signal from two covariates removed from the risk score. Future work should adopt a time-varying coefficient extension to obtain fully valid hazard ratio estimates for Hippocampus_*σ*_ and Ventricles_*σ*_. Fourth, the 11 significant covariates in the Cox PH model (Table 7) were identified without correction for multiple comparisons across 24 tests; approximately 1–2 false positives are expected by chance at p<0.05, and reported hazard ratios should be interpreted as exploratory rather than confirmatory. Fifth, death before conversion is a non-trivial competing risk in a cohort with mean age 72.7 years; treating it as non-informative censoring under the Kaplan-Meier and Cox frameworks may overestimate the cumulative incidence of conversion. A formal Fine-Gray subdistribution hazard analysis could not be performed on ADNI because death records are not directly coded in ADNIMERGE (the variable DX takes only the values CN, MCI, and Dementia). Fine-Gray competing-risk analysis is a primary analytical objective of the planned NACC validation study, where cause-of-exit data are available under the Uniform Data Set (UDS) protocol; this constitutes a committed next step rather than an open research question. Future extensions should also incorporate competing-risk decomposition of the Brier score, which jointly accounts for death and non-AD dementia as distinct absorbing states. Sixth, the benchmark does not include a nested cross-validation with hyperparameter search for the deep learning models; DeepSurv and DeepHit are known to be sensitive to architecture and learning-rate choices, and the use of published defaults may underestimate their maximum discriminative potential relative to the classical models, which are substantially more robust to the choice of regularisation parameter. Seventh, the three scalar descriptors per region offer an efficient but compressed representation; attention-based sequence encoders or functional data analysis methods may recover additional prognostic signal for patients with non-linear or accelerating atrophy patterns. Eighth, a difference in the minimum MRI session criterion between ADNI (≥3 sessions) and OASIS-3 (≥2 sessions) introduces an asymmetry in the temporal descriptor reliability across cohorts. A sensitivity analysis reducing the ADNI threshold to ≥2 sessions (reported in Section 3.2) confirmed that the main results are robust: the Cox PH C-index changed by Δ=−0.005 (0.858±0.030 vs. 0.863±0.027), with a modest increase in fold-to-fold variability consistent with noisier two-point slope estimates.

## 6. Conclusions

This study introduced a temporal MRI descriptor pipeline for time-to-event prediction in MCI, combining atrophy rate, trajectory variability, and time-weighted cumulative mean across six brain regions with standard clinical variables. Applied across seven survival model families, the pipeline achieves a C-index of 0.863 using only routine structural MRI without PET, CSF, or multimodal data, demonstrating strong discriminative performance for MCI-to-AD conversion prediction. The result is consistent across all seven model families, in line with published meta-analyses at comparable sample sizes. Ventricular expansion slope and ADAS13 emerged as the dominant risk amplifiers, and all cortical atrophy slopes showed protective effects consistent with established AD neuroanatomy, confirming that capturing dynamic volumetric trajectories adds prognostic value beyond static cognitive scores.

Exploratory validation on an independent OASIS-3 cohort (n=78, EPV ≈ 1 per fold) revealed a preliminary stratification by model family: classical and regularised models maintained higher C-index with lower fold-to-fold variability, while deep learning architectures degraded substantially under data-scarce conditions and covariate distribution shift. Although the small per-fold sample size prevents confirmatory conclusions, these preliminary results are directionally consistent with the hypothesis that Cox PH and Weibull AFT show more stable cross-cohort performance under distribution shift, offering interpretable hazard ratios and no GPU requirements. Confirmatory validation on NACC (n>40,000, full covariate parity including ADAS-13) and competing-risk modelling (Fine-Gray, accounting for death before conversion) constitute the primary directions for consolidating these findings.

## Figures and Tables

**Figure 1:**
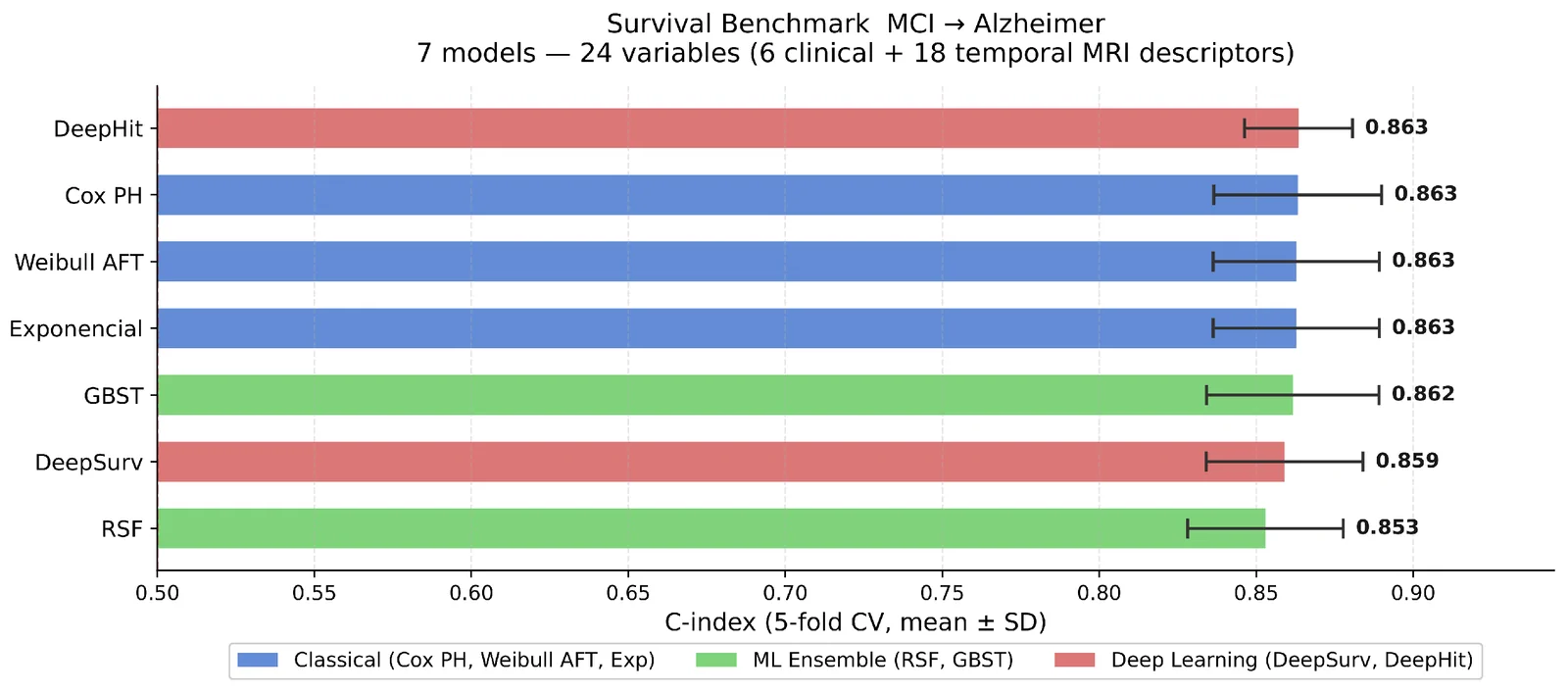
C-index comparison across seven survival models (5-fold CV, n=661, 24 variables). All models converge within a narrow band (Δ=0.010), with no clear performance separation among architectures in this setting. Error bars: ±1 SD. Dashed line: random-classifier baseline (C=0.50).

**Figure 2:**
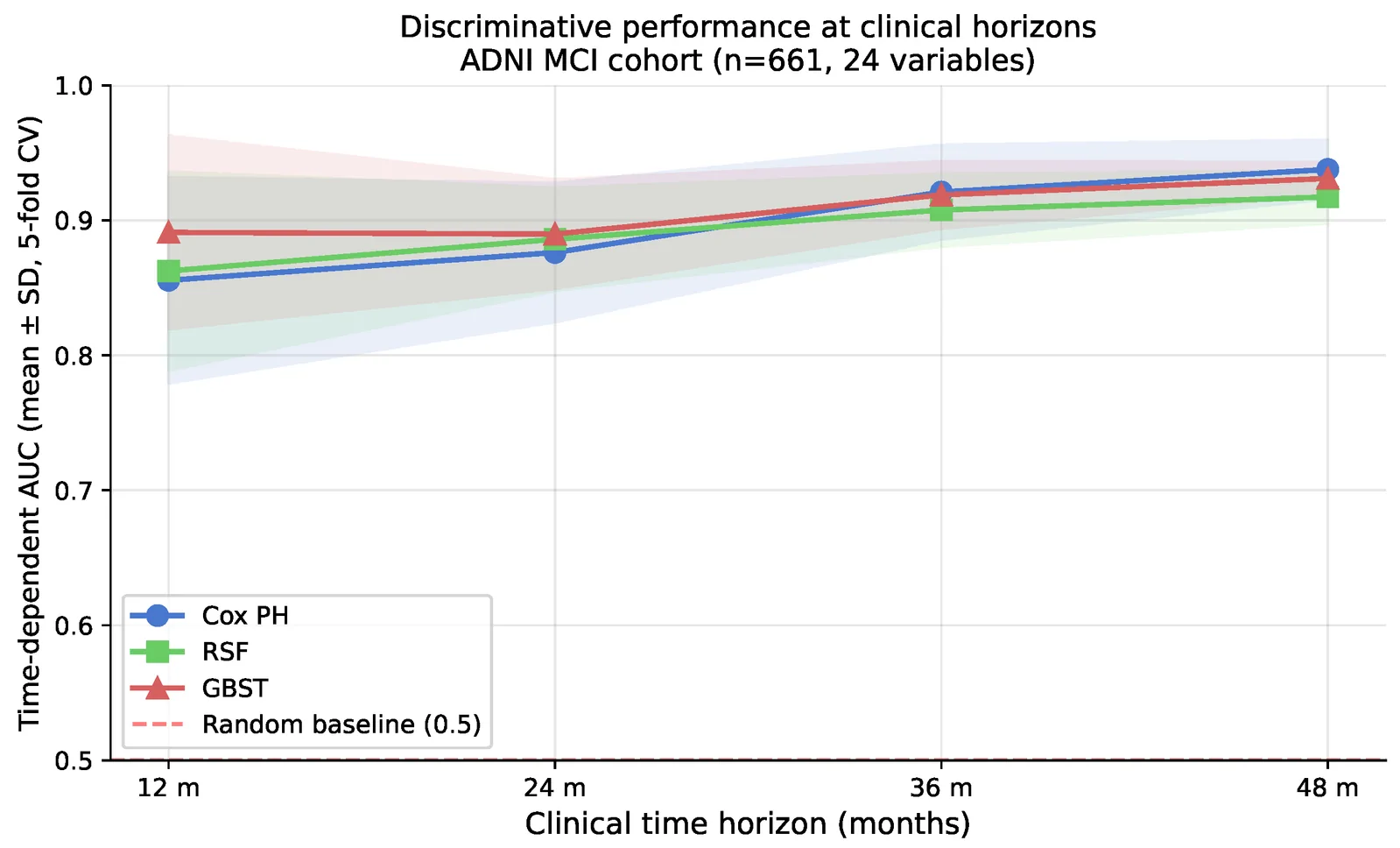
Time-dependent AUC at clinical prognostic horizons (12, 24, 36, and 48 months) for Cox PH, RSF, and GBST (mean ± SD, 5-fold CV, n=661). The dashed red line marks the random-classifier baseline (AUC = 0.5). All models maintain AUC > 0.84 through 48 months, confirming sustained discriminative utility across the full follow-up window of the ADNI cohort.

**Figure 3:**
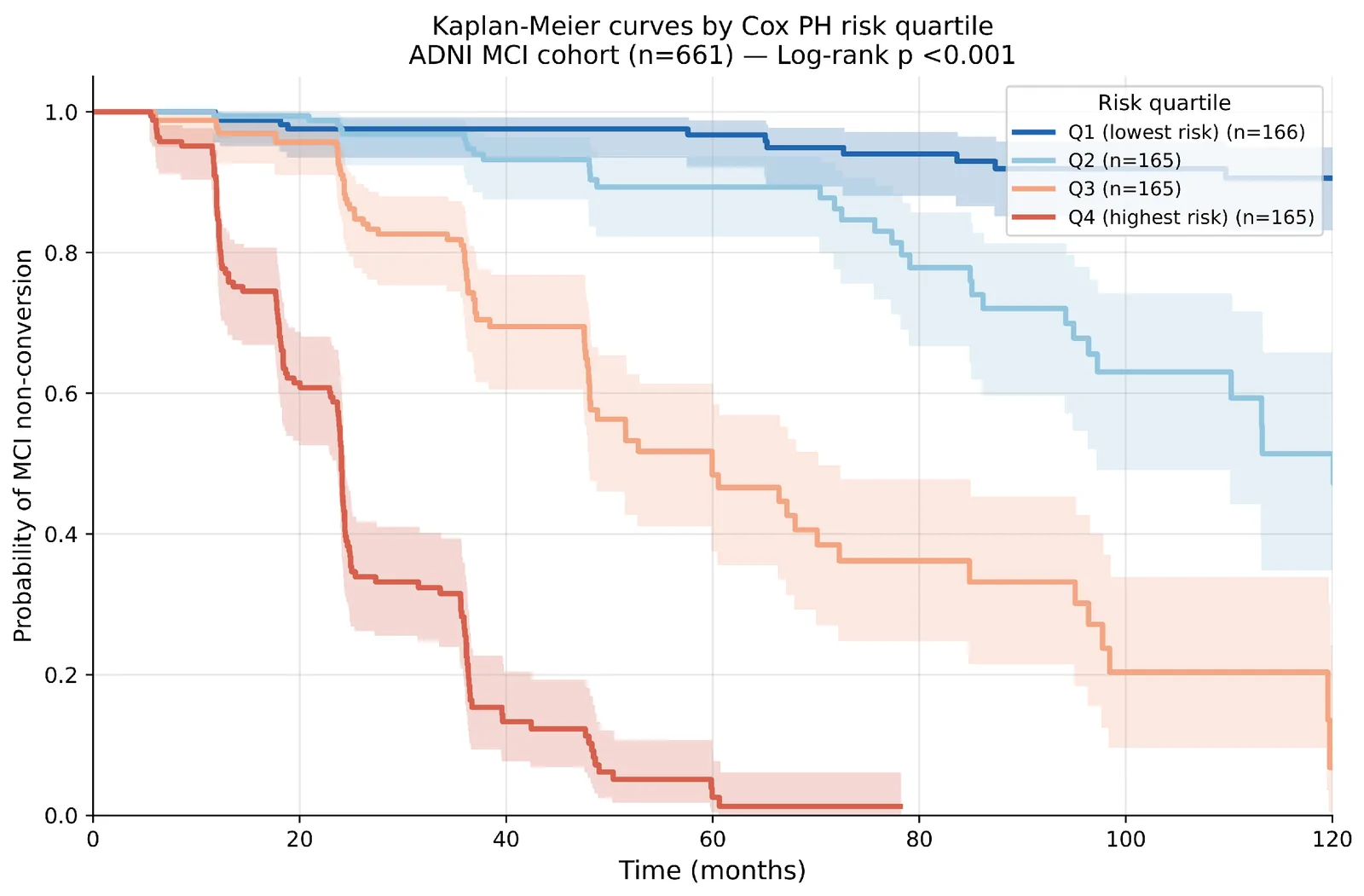
Kaplan–Meier curves for MCI non-conversion probability by Cox PH risk quartile (Q1 = lowest risk, Q4 = highest risk) on the full ADNI cohort (n=661). Log-rank p<0.001 across quartiles. Shaded areas represent 95% confidence intervals. Patients in Q4 convert at substantially earlier times than Q1 patients, demonstrating clinically meaningful risk stratification by the temporal descriptor pipeline.

**Figure 4:**
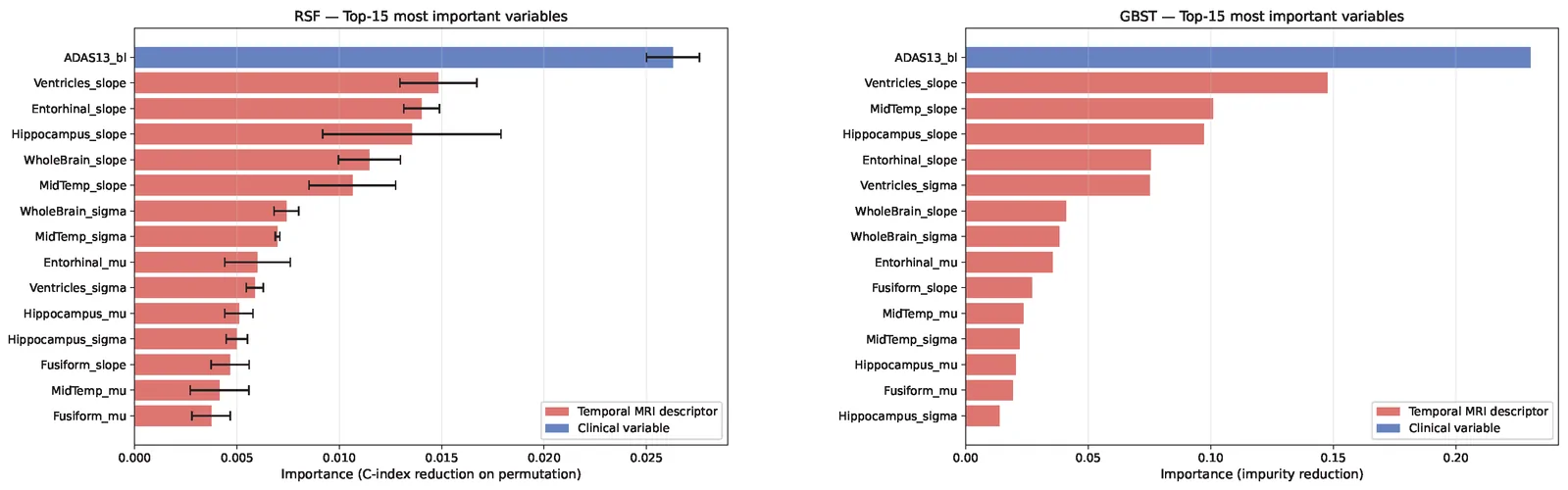
Variable importance for the two ensemble models. (**Left**): RSF permutation importance (decrease in C-index when a covariate is randomly permuted out-of-bag). (**Right**): GBST total gradient contribution per covariate. ADAS13 and ventricular expansion slope rank highest in both methods; temporal MRI descriptors dominate over static clinical variables, confirming the added value of serial neuroimaging features.

**Figure 5:**
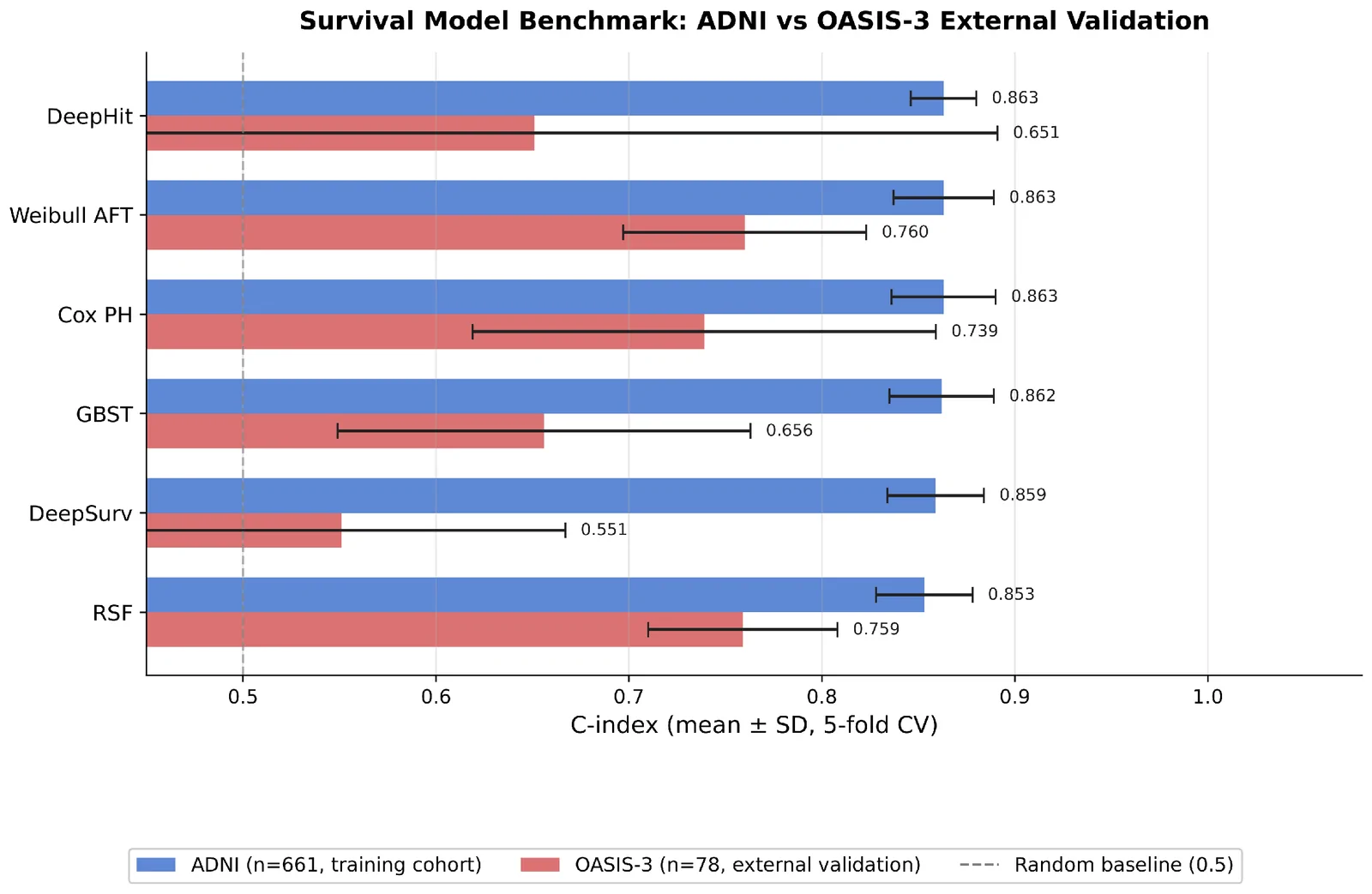
Exploratory cross-cohort C-index comparison between ADNI (n=661, blue) and OASIS-3 (n=78, red; hypothesis-generating only, EPV ≈ 1). Classical models show more stable cross-cohort behaviour than deep learning and ensemble models. Error bars: ±1 SD. Dashed line: random-classifier baseline (C=0.50).

**Table 1:** Hyperparameter configuration for all survival models. No automated search was performed; values follow published defaults or standard regularisation conventions for the dataset size (n=661, p=24).

Model	Parameter	Value
Cox PH	L2 penalty (α)	0.1
Weibull AFT	L2 penalty (α)	0.1
RSF	Trees (*B*)	200
	Min. terminal node size	10 events
	Features per split (*m*)	$\left\lfloor \sqrt{p}\right\rfloor$
GBST	Estimators (*M*)	200
	Learning rate (η)	0.05
	Max. tree depth (*d*)	3
DeepSurv	Architecture	MLP [64,64], BatchNorm, ReLU
	Dropout	p=0.2
	Optimizer	Adam, $lr=10^{-3}$
	Training	100 epochs, batch size 64
DeepHit	Architecture	MLP [64,64], BatchNorm, ReLU
	Dropout	p=0.2
	Optimizer	Adam, $lr=5\times 10^{-4}$
	Loss weights	α=0.5, σ=0.1
	Time discretisation	K=20 quantile bins
	Early stopping	patience 20, up to 300 epochs
	Batch size	64

**Table 2:** Cohort characteristics (mean ± SD or *n* (%)).

Variable	Primary (*n* = 993)	Analytical (*n* = 661)
Age (years)	73.0±7.5	72.2±7.4
Sex (male)	589 (59.3%)	388 (58.7%)
Education (years)	16.0±2.8	16.0±2.8
APOE4 = 0	501 (50.5%)	329 (49.8%)
APOE4 = 1	385 (38.8%)	258 (39.0%)
APOE4 = 2	107 (10.8%)	74 (11.2%)
MMSE	27.6±1.8	27.7±1.8
ADAS-13	16.4±6.8	15.7±6.6
Conversion events	376 (37.9%)	250 (37.8%)
Median follow-up (months)	35.9	42.0

**Table 3:** Baseline characteristics of included (n=661) versus excluded (n=332) participants. Continuous variables compared with the Mann–Whitney *U* test; categorical variables with the $\chi^{2}$ test.

Variable	Included (*n* = 661)	Excluded (*n* = 332)	*p*-Value
Age (years)	72.2±7.4	74.5±7.5	<0.001
Sex (male)	388 (58.7%)	201 (60.5%)	0.625
Education (years)	16.0±2.8	15.9±2.8	0.374
MMSE	27.7±1.8	27.3±1.9	0.002
ADAS-13	15.7±6.6	17.9±6.9	<0.001
APOE4 = 0	329 (49.8%)	172 (51.8%)	0.591
APOE4 = 1	258 (39.0%)	127 (38.3%)	0.866
APOE4 = 2	74 (11.2%)	33 (9.9%)	0.622
Conversion rate	250 (37.8%)	126 (38.0%)	1.000
Mean follow-up (months)	54.4±37.9	24.5±20.2	<0.001

**Table 4:** Feature-set ablation using Cox PH (n=661, 5-fold CV). A1: clinical only; A2: clinical + baseline MRI (first visit, ICV-normalised); A3: clinical + temporal MRI descriptors (full pipeline). C-index reported as mean ± SD.

Configuration	Features	C-Index (Mean ± SD)	Δ vs. Previous
A1: Clinical only	6	0.794±0.035	—
A2: Clinical + Baseline MRI	12	0.814±0.022	+0.021
A3: Clinical + Temporal MRI	24	0.863±0.027	+0.049

**Table 5:** Survival model benchmark: 5-fold CV C-index and Integrated Brier Score (IBS, mean ± SD) on the analytical cohort (*n* = 661, 24 variables: 6 clinical + 18 temporal MRI descriptors). IBS evaluated at percentiles 10–90% of observed event times (15 grid points, range 12–80 months). Lower IBS indicates better calibration; null-model IBS = 0.25.

Model	Family	CV C-index	IBS
Cox PH	Classical	0.863±0.027	0.084±0.012
Weibull AFT	Classical	0.863±0.026	0.085±0.011
Exponential	Classical	0.863±0.026	— ^*b*^
GBST	ML Ensemble	0.862±0.027	0.089±0.010
RSF	ML Ensemble	0.853±0.025	0.100±0.008
DeepSurv	Deep Learning	0.859±0.025	— ^*a*^
DeepHit	Deep Learning	0.863±0.017	0.098±0.005

^*a*^ IBS is not reported for DeepSurv: in 4 of 5 folds, the Breslow baseline hazard estimator returned a numerically flat cumulative hazard after 100 fixed training epochs, making survival probability outputs degenerate for IBS computation. The value from the single valid fold is not reported to avoid a misleading single-fold comparison. The C-index is unaffected because it depends only on the scalar risk score $\phi (z_{i};\theta )$, which remained well-defined in all 5 folds. ^*b*^ IBS is not reported for the Exponential model: the lifelines implementation does not expose a per-observation survival function at arbitrary grid times in the same API used for Cox PH and Weibull AFT, making IBS computation unavailable without custom numerical integration; the C-index is unaffected.

**Table 6:** Time-dependent AUC (mean ± SD, 5-fold CV) at clinical prognostic horizons on the ADNI cohort (*n* = 661), estimated following the inverse-probability-of-censoring-weighted estimator of Uno et al. [22]; AUC = 0.5 indicates random discrimination. Cox PH, RSF, and GBST are reported as representative models of the three methodological families (classical, ensemble, and gradient boosting); the remaining four models showed equivalent or lower discrimination across all horizons.

Model	12 Months	24 Months	36 Months	48 Months
Cox PH	0.856±0.076	0.876±0.051	0.921±0.035	0.938±0.022
GBST	0.891±0.072	0.890±0.040	0.919±0.025	0.931±0.012
RSF	0.862±0.074	0.886±0.038	0.908±0.027	0.917±0.020

**Table 7:** Covariate effects in the Cox PH model (*n* = 661, 24 variables, L2 α=0.1) for variables attaining p<0.05 (raw Wald test). HR: hazard ratio; IC 95%: Wald confidence interval; *q*: Benjamini–Hochberg FDR-adjusted *p*-value (computed over all 24 covariates). HR > 1: increased conversion risk; HR < 1: protective effect. Results are exploratory; ^†^ denotes q≥0.05 (does not survive FDR correction). *** p<0.001, ** p<0.01, * p<0.05.

Variable	HR	IC 95%	Sig.	*q*
ADAS13	1.616	[1.409, 1.854]	***	<0.001
APOE4	1.206	[1.077, 1.350]	**	0.006
Slope Ventricles	1.420	[1.232, 1.636]	***	<0.001
Slope MidTemp	0.782	[0.681, 0.898]	***	0.003
Slope Hippocampus	0.801	[0.692, 0.927]	**	0.011
Slope WholeBrain	0.808	[0.701, 0.933]	**	0.012
Slope Entorhinal	0.838	[0.738, 0.952]	**	0.020
Slope Fusiform	0.846	[0.737, 0.971]	*	0.042
σ Ventricles	0.702	[0.604, 0.815]	***	<0.001
σ MidTemp	0.815	[0.689, 0.965]	*	0.042
σ WholeBrain	0.814	[0.672, 0.985]	*^†^	0.076

**Table 8:** Top-5 VIF values among the 24 covariates. All 24 VIF <5 (no multicollinearity).

Covariate	VIF
σ WholeBrain	4.81
σ MidTemp	4.65
μ WholeBrain	3.97
σ Fusiform	3.85
μ Hippocampus	3.63
Median (all 24)	2.33
Maximum	4.81

**Table 9:** Exploratory independent-cohort analysis on OASIS-3: 5-fold CV C-index (mean ± SD) for six survival models (*n* = 78, 23 variables, no ADAS-13). ADNI results reproduced from Table 5 for comparison. Δ = ADNI minus OASIS-3 mean C-index.

Model	Family	ADNI (*n* = 661)	OASIS-3 (*n* = 78)	Δ
Cox PH	Classical	0.863±0.027	0.739±0.120	−0.124
Weibull AFT	Classical	0.863±0.026	0.760±0.063	−0.103
RSF	ML Ensemble	0.853±0.025	0.759±0.049	−0.094
GBST	ML Ensemble	0.862±0.027	0.656±0.107	−0.206
DeepSurv	Deep Learning	0.859±0.025	0.551±0.116	−0.308
DeepHit	Deep Learning	0.863±0.017	0.651±0.240	−0.212

## Data Availability

The primary data analyzed in this study were obtained from the Alzheimer’s Disease Neuroimaging Initiative (ADNI) database (https://adni.loni.usc.edu, accessed on 15 January 2025), available to registered investigators. Data for the exploratory cross-cohort analysis were obtained from the OASIS-3 dataset (https://www.oasis-brains.org, accessed on 15 January 2025), available under a Data Use Agreement to registered investigators. Analysis code is openly available at https://github.com/NathaliaArias/adni-survival-benchmark (accessed on 15 January 2025) under an MIT licence; a Zenodo archive with a citable DOI will be registered upon acceptance.
